# A Novel CCS-MPC Algorithm for SPMSM Position Control with Innovative Constraint Processing Strategy

**DOI:** 10.3390/s26051527

**Published:** 2026-02-28

**Authors:** Lingbo Kong, Jianli Wang, Xinyu Dong, Huiying Hu, Boting Liu

**Affiliations:** 1 Changchun Institute of Optics, Fine Mechanics and Physics, Chinese Academy of Sciences, Changchun 130000, China; konglingbo@jl1.cn; 2 The University of Chinese Academy of Sciences, Changchun 130000, China; 3 Chang Guang Satellite Technology Co., Ltd., Changchun 130000, China; dongxinyu1017@jl1.cn (X.D.); huhuiying0628@jl1.cn (H.H.); liuboting@jl1.cn (B.L.)

**Keywords:** model predictive position control (MPPC), surface-mounted PMSM (SPMSM), reference trajectory planning

## Abstract

In this study, we develop a novel continuous control set model predictive control (NCCS-MPC) strategy for surface-mounted PMSM (SPMSM) position control, which consists of the improved multi-step model predictive position control (IMS-MPPC) algorithm and an innovative constraint processing strategy (ICPS). The algorithm reforms the predictive model in the conventional strategy for multi-step model predictive position control, and the one-step computational delay is compensated for within the multi-step CCS-MPC frame, guaranteeing steady-state performance. The ICPS takes speed and current constraints into account during reference trajectory planning and imposes a voltage constraint on the unconstrained optimization result, removing the problem of constraint conflict. A comparative simulation study combined with experimental results verified the merits of the proposed NCCS-MPC.

## 1. Introduction

Surface-mounted permanent magnet synchronous motors (SPMSMs) have been widely used in industrial fields such as robotics [1], aerospace [2], and electric vehicles [3] due to their outstanding advantages of high power density, high reliability, and good control performance. The classical control method for SPMSMs is field-oriented-based cascaded PID control, which has the advantages of simplicity and good robustness. However, the SPMSM is a typical nonlinear, coupled system, and with PID control, it is difficult to achieve a fast response, small overshoot, and high steady-state accuracy simultaneously. Therefore, in high-end applications, advanced control algorithms such as sliding mode control [4], adaptive control [5], backstepping control [6], and model predictive control (MPC) [7,8] have been widely studied, and many achievements have been made in improving the control performance of SPMSMs. Among these algorithms, MPC has received increasing attention due to the merits of intuitive modeling and tuning, easy inclusion of nonlinearity and constraints, and multiple control objectives.

MPC is classified as finite control set model predictive control (CCS-MPC) or continuous set model predictive control (FCS-MPC), based on whether it solves an integer optimization problem [9]. FCS-MPC directly considers the discrete characteristics of power converters and uses exhaustive methods to evaluate the control effect of the eight basic voltage vectors. The voltage vector that minimizes the cost function is selected as the output of the power converter in the next control cycle [10,11,12]. CCS-MPC obtains continuous control signals by solving quadratic optimization problems [13] and then uses PWM technology (mostly SVPWM) to modulate the optimal control signal. In the field of motor control, MPC is more commonly used for direct current (torquer) control [14,15,16,17], but relatively less studied in the high-order position control or speed control system.

However, cascaded-free direct position control based on the MPC algorithm performs better in dynamic response than the conventional PID strategy does [8]. Reference [18] presented an FCS-MPC-based direct position control approach, where good track performance was demonstrated, but only one voltage vector was applied during a complete switching cycle in the FCS-MPC frame, and achieving system steady-state performance was challenging because of high current ripples. The steady-state ripple is alleviated to some extent via duty-ratio and multi-voltage vector technology in [19]. Moreover, the FCS-MPC method is commonly employed in short predictive horizon problems [9] combined with the deadbeat principle [20] where different sample times between the position and current are necessary because the electromagnetic time constant is much smaller than the mechanical time constant.

Compared to the FCS-MPC method, CCS-MPC can perform better in the steady state because of the modulation technology. In addition, the CCS-MPC method is flexible in handling long-predictive-horizon problems, which is important for high-order control systems to achieve better stability and performance. S.D. Huang et al. applied the CCS-MPC method in predictive position control for a long-stroke planar motor [21] and achieved micrometer-level control accuracy. Mohammadreza Mamashli et al. designed speed and current loops by adopting a method that combines MPC and sliding mode control, effectively reducing torque ripple and losses [22]. However, the main challenge of implementing the CCS-MPC algorithm is determining how to solve the quadratic optimization problem effectively, even with conflict among the constraints. The constraints in the position control system for SPMSM usually include the speed, current, and voltage limits [20]. The speed and current constraints concern the long-term safe operation of the system, while limits can be exceeded for short periods with small amplitudes, commonly referred to as soft constraints [23]; the voltage constraint is determined by the DC-Link voltage and modulation method and must be strictly respected, commonly known as hard constraints [23]. Hildreth’s quadratic planning procedure [13,24] is the most used method in CCS-MPC optimization, but it treats both types of constraints as soft. When conflict occurs, the convergence and voltage limit cannot be guaranteed, so it is difficult for the actual system performance to remain optimal. C. He et al. proposed a geometric optimization method in [25] and showed that it is more effective than Hildreth’s algorithm. Unfortunately, this method treats all the constraints as hard, leading to the so-called deadlock during conflict.

Besides the optimization problem mentioned above, the inherent one-step computational delay phenomenon that exists in the digital control system is not treated well in the long-horizon multi-step CCS-MPC algorithm in [8,21,25,26,27,28,29,30], causing chattering in the steady-state response [31].

**Figure 1:**
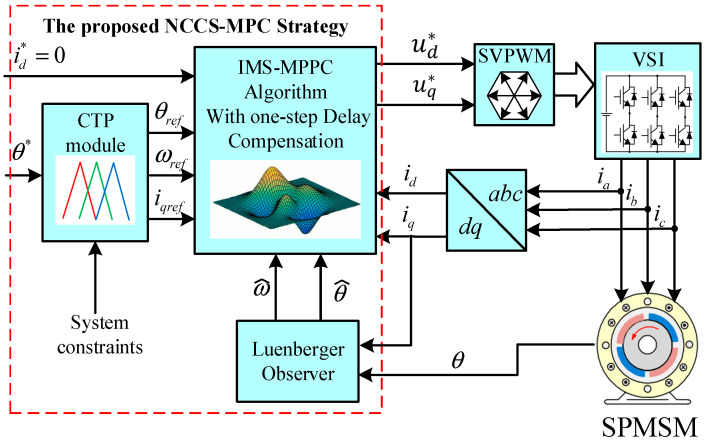
The control diagram of the proposed NCCS-MPC strategy.

In this study, a novel CCS-MPC strategy (NCCS-MPC) for surface-mounted PMSM (SPMSM) position control is developed, which consists of the improved multi-step model predictive position control algorithm (IMS-MPPC) and an innovative constraint processing strategy (ICPS). The control diagram of the proposed NCCS-MPC strategy is illustrated in Figure 1. The constrained trajectory planner (CTP) is the core of the ICPS and produces the constraint-compatible reference trajectories of stator current, angular speed, and angular position. These trajectories are then fed into the IMS-MPPC module to construct the cost function. The main contributions of this paper can be summarized as follows:

(1) The proposed IMS-MPPC algorithm reforms the predictive model in the conventional strategy for multi-step model predictive position control. The strategy of one-step delay compensation is used for the first time in the CCS-MPC framework based on multi-step prediction on SPMSM.
(2) The proposed ICPS adopts distinct disposals for system constraints, concerning their different properties. Speed and current limits are considered in the CTP module to plan the constraint-compatible trajectories for the IMS-MPPC to track, so the constrained problem degrades to an unconstrained one and the unconstrained optimal solution is further limited to the voltage vector circle to respect the voltage constraints. The ICPS balances the hard and soft constraints well, and deadlock and non-convergence are consequently avoided.

This paper is organized as follows. Section 2 introduces the proposed IMS-MPPC algorithm with its predictive model and cost function for SPMSM position control. The proposed ICPS is described in Section 3 in detail regarding how it deals with the system constraints. Section 4 briefly describes the design of the extended Luenberger observer in SPMSM. Section 5 demonstrates the simulation and experimental results to validate the advantages of the proposed NCCS-MPC over other MPC strategies, and finally, Section 6 concludes the paper.

## 2. The Proposed IMS-MPPC Algorithm

### 2.1. Full-Order Model of SPMSM

The dynamic model of SPMSM in the d and q synchronous rotating coordinate system is described as follows:

(1) $$\frac{di_{d}}{dt}=\frac{1}{L_{s}}\left(u_{d}-R_{s}i_{d}+\omega_{e}L_{s}i_{q}\right)$$

(2) $$\frac{di_{q}}{dt}=\frac{1}{L_{s}}\left(u_{q}-R_{s}i_{q}-\omega_{e}L_{s}i_{d}-\omega_{e}\psi_{f}\right)$$

(3) $$\frac{d\omega_{e}}{dt}=\frac{3p^{2}\psi_{f}}{2J}i_{q}-\frac{p}{J}T_{L}$$

(4) $$\frac{d\theta_{e}}{dt}=\omega_{e}$$

where $u_{d}$, $u_{q}$, $i_{d}$, and $i_{q}$ are the d- and q-axis voltages and currents, respectively, and $\theta_{e}$ and $\omega_{e}$ correspond to the electrical angular position and speed. The inductance and resistance of the *d*- and *q*-axes are basically equal in actual measurement, so are assumed equal for the convenience of research. $L_{s}$ and $R_{s}$ are the excitation inductance and resistance of the motor stator, respectively, and $\psi_{f}$, p, J, and $T_{L}$ represent the permanent magnet flux linkage, pole pairs, moment of inertia, and load torque, respectively.

### 2.2. Discrete State Space of SPMSM

The dynamics in Equations (1)–(4) are discretized using 1st-, 2nd-, and 3rd-order Taylor expansions, and ${\dot{T}}_{L}=0$ is supposed owing to the high system sampling frequency.

(5) $$i_{d}\left(k+1\right)=i_{d}\left(k\right)+\frac{T_{s}}{L_{s}}\left(u_{d}\left(k\right)-R_{s}i_{d}\left(k\right)+\omega_{e}\left(k\right)L_{s}i_{q}\left(k\right)\right)$$

(6) $$i_{q}\left(k+1\right)=i_{q}\left(k\right)+\frac{T_{s}}{L_{s}}\left(u_{q}\left(k\right)-R_{s}i_{q}\left(k\right)-\omega_{e}\left(k\right)L_{s}i_{d}\left(k\right)-\psi_{f}\omega_{e}\left(k\right)\right)$$

(7) $$\omega_{e}\left(k+1\right)=\omega_{e}\left(k\right)+T_{s}\left(\frac{3p^{2}\psi_{f}}{2J}i_{q}\left(k\right)-\frac{p}{J}T_{L}\left(k\right)\right)+\frac{T_{s}^{2}}{2}\frac{3p^{2}\psi_{f}}{2J}\frac{1}{L_{s}}\left(u_{q}\left(k\right)-R_{s}i_{q}\left(k\right)-\omega_{e}\left(k\right)L_{s}i_{d}\left(k\right)-\omega_{e}\left(k\right)\psi_{f}\right)$$

(8) $$\theta_{e}\left(k+1\right)=\theta_{e}\left(k\right)+T_{s}\omega_{e}\left(k\right)+\frac{T_{s}^{2}}{2}\left(\frac{3p^{2}\psi_{f}}{2J}i_{q}\left(k\right)-\frac{p}{J}T_{L}\left(k\right)\right)+\frac{T_{s}^{3}}{6}\frac{3p^{2}\psi_{f}}{2J}\frac{1}{L_{s}}\left(u_{q}\left(k\right)-R_{s}i_{q}\left(k\right)-\omega_{e}\left(k\right)L_{s}i_{d}\left(k\right)-\omega_{e}\left(k\right)\psi_{f}\right)$$

$T_{s}$ represents the sample period. The method in [19] is used to modify Equations (5) and (6) to improve the current predictive accuracy as

(9) $$i_{d}\left(k+1\right)=i_{d}\left(k\right)+\frac{T_{s}}{L_{s}}\left(1-\frac{T_{s}R_{s}}{2L_{s}}\right)\left(u_{d}\left(k\right)-R_{s}i_{d}\left(k\right)+\omega_{e}\left(k\right)L_{s}i_{q}\left(k\right)\right)$$

(10) $$i_{q}\left(k+1\right)=i_{q}\left(k\right)+\frac{T_{s}}{L_{s}}\left(1-\frac{T_{s}R_{s}}{2L_{s}}\right)\left(u_{q}\left(k\right)-R_{s}i_{q}\left(k\right)-\omega_{e}\left(k\right)L_{s}i_{d}\left(k\right)-\psi_{f}\omega_{e}\left(k\right)\right)$$

The system output vector and state vector are defined as $y_{m}\left(k\right)=x_{m}\left(k\right)={\left[\begin{array}{cccc} i_{d}\left(k\right) & i_{q}\left(k\right) & \omega_{e}\left(k\right) & \theta_{e}\left(k\right) \end{array}\right]}^{T}$, and the control input is defined as $u_{m}\left(k\right)=\left[\begin{array}{c} u_{md}\left(k\right)\\ u_{mq}\left(k\right) \end{array}\right]=\left[\begin{array}{c} u_{d}\left(k\right)+\omega_{e}\left(k\right)L_{s}i_{q}\left(k\right)\\ u_{q}\left(k\right)-\omega_{e}\left(k\right)L_{s}i_{d}\left(k\right) \end{array}\right]$; then, the discrete state space of the position control system of SPMSM is formulated as

(11) $$x_{m}\left(k+1\right)=A_{m}x_{m}\left(k\right)+B_{m}\left(k\right)u_{m}\left(k\right)+E_{m}\left(k\right)$$

(12) $$y_{m}\left(k\right)=C_{m}x_{m}\left(k\right)$$

where $A_{m}=[\begin{array}{cc} 1-\frac{T_{s}R_{s}}{L_{s}}\left(1-\frac{T_{s}R_{s}}{2L_{s}}\right) & 0\\ 0 & 1-\frac{T_{s}R_{s}}{L_{s}}\left(1-\frac{T_{s}R_{s}}{2L_{s}}\right)\\ 0 & \frac{3p^{2}T_{s}\psi_{f}\left(2L_{s}-T_{s}R_{s}\right)}{4JL_{s}}\\ 0 & \frac{p^{2}T_{s}^{2}\psi_{f}\left(3L_{s}-T_{s}R_{s}\right)}{4JL_{s}} \end{array}\;\begin{array}{cc} 0 & 0\\ -\frac{T_{s}\psi_{f}}{L_{s}}\left(1-\frac{T_{s}R_{s}}{2L_{s}}\right) & 0\\ 1-\frac{3T_{s}^{2}p^{2}\psi_{f}^{2}}{4JL_{s}} & 0\\ T_{s}-\frac{p^{2}T_{s}^{3}\psi_{f}^{2}}{4JL_{s}} & 1 \end{array}]$, $B_{m}=\left[\begin{array}{cc} \frac{T_{s}}{L_{s}}\left(1-\frac{T_{s}R_{s}}{2L_{s}}\right) & 0\\ 0 & \frac{T_{s}}{L_{s}}\left(1-\frac{T_{s}R_{s}}{2L_{s}}\right)\\ 0 & \frac{3p^{2}T_{s}^{2}\psi_{f}}{4JL_{s}}\\ 0 & \frac{p^{2}T_{s}^{3}\psi_{f}}{4JL_{s}} \end{array}\right]$, $C_{m}=I_{4\times 4}$, and $E_{m}\left(k\right)={\left[\begin{array}{cccc} 0 & 0 & -\frac{T_{s}pT_{L}\left(k\right)}{J} & -\frac{T_{s}^{2}pT_{L}\left(k\right)}{2J} \end{array}\right]}^{T}$ is the disturbance term.

### 2.3. The Proposed IMS-MPPC Strategy

Neglecting the variation in disturbance term $E_{m}$ between two consecutive samples, the incremental state space of the system is expressed as

(13) $$\Delta x_{m}\left(k+1\right)=A_{m}\Delta x_{m}\left(k\right)+B_{m}\left(k\right)\Delta u_{m}\left(k\right)$$

(14) $$y_{m}\left(k+1\right)=y_{m}\left(k\right)+C_{m}A_{m}\Delta x_{m}\left(k\right)+C_{m}B_{m}\left(k\right)\Delta u_{m}\left(k\right)$$

The new state vector and output vector are defined as $x\left(k\right)={\left[\begin{array}{cc} \Delta x_{m}^{T}\left(k\right) & y_{m}^{T}\left(k\right) \end{array}\right]}^{T}$ and $y\left(k\right)=y_{m}\left(k\right)$, and then the new state space is described as

(15) $$x\left(k+1\right)=A_{0}x\left(k\right)+B_{0}\Delta u_{m}\left(k\right)$$

(16) $$y\left(k\right)=C_{0}x\left(k\right)$$

where $A_{0}=\left[\begin{array}{cc} A_{m} & 0_{4\times 4}\\ C_{m}A_{m} & I_{4\times 4} \end{array}\right]$, $B_{0}=\left[\begin{array}{c} B_{m}\\ C_{m}B_{m} \end{array}\right]$, $C_{0}=\left[\begin{array}{cc} 0_{4\times 4} & I_{4\times 4} \end{array}\right]$, and $∆u_{m}\left(k\right)=\left[\begin{array}{c} ∆u_{d}\left(k\right)+\omega_{e}\left(k\right)L_{s}i_{q}\left(k\right)-\omega_{e}\left(k-1\right)L_{s}i_{q}\left(k-1\right)\\ ∆u_{q}\left(k\right)-\omega_{e}\left(k\right)L_{s}i_{d}\left(k\right)+\omega_{e}\left(k-1\right)L_{s}i_{d}\left(k-1\right) \end{array}\right]$, and $0_{4\times 4}$ and $I_{4\times 4}$, respectively, denote the zero and identity matrices of compatible dimensions. When considering the control horizon $N_{c}$ and the prediction horizon $N_{p}$ ($N_{c}$ ≤ $N_{p}$), the following vectors are defined:

$$Y\left(k+1\right)={\left[\begin{array}{ccc} {y\left(k+1\right)}^{T} & {y\left(k+2\right)}^{T} & \begin{array}{cc} \cdots & {y\left(k+N_{p}\right)}^{T} \end{array} \end{array}\right]}^{T}$$

$$\Delta U\left(k\right)={\left[\begin{array}{ccc} {\Delta u_{m}\left(k\right)}^{T} & \begin{array}{c} \cdots \end{array} & {\Delta u_{m}\left(k+N_{c}-1\right)}^{T} \end{array}\right]}^{T}$$

The system state predictive model for the future $N_{p}$ samples can be established as

(17) Y(k+1)=Fx(k)+GΔU(k)

where $F=\left[\begin{array}{c} C_{0}A_{0}\\ C_{0}A_{0}^{2}\\ \begin{array}{c} \vdots \\ C_{0}A_{0}^{N_{p}} \end{array} \end{array}\right]$, $G=\left[\begin{array}{cccc} C_{0}B_{0} & 0 & \ldots & 0\\ C_{0}A_{0}B_{0} & C_{0}B_{0} & \ldots & 0\\ \vdots & \vdots & \ddots & \vdots \\ C_{0}A_{0}^{N_{p}-1}B_{0} & C_{0}A_{0}^{N_{p}-2}B_{0} & \ldots & C_{0}A_{0}^{N_{p}-N_{c}}B_{0} \end{array}\right]$.

However, there is a one-step delay in digital control systems. When the optimal control sequence is obtained according to the predictive model (17) and the first sample ${\Delta u_{m}\left(k\right)}^{T}$ is output to the modulation module, it will not act on the motor until the (k+1)th sampling period, and will consequently lead to chattering [28]. To compensate for the one-step delay phenomenon, (17) is reformed as

(18) $$Y\left(k+1\right)=\bar{F}\bar{x}\left(k\right)+\bar{G}\Delta U\left(k+1\right)$$

where $\bar{F}=\left[\begin{array}{cc} C_{0}A_{0} & C_{0}B_{0}\\ C_{0}A_{0}^{2} & C_{0}A_{0}B_{0}\\ \vdots & \vdots \\ C_{0}A_{0}^{N_{p}} & C_{0}A_{0}^{N_{p}-1}B_{0} \end{array}\right]$, $\bar{x}\left(k\right)={\left[\begin{array}{cc} x^{T}\left(k\right) & ∆u_{m}^{T}\left(k\right) \end{array}\right]}^{T}$, $\bar{G}=\left[\begin{array}{ccc} 0 & \ldots & 0\\ C_{0}B_{0} & \ldots & 0\\ \vdots & \ddots & \vdots \\ C_{0}A_{0}^{N_{p}-2}B_{0} & \ldots & C_{0}A_{0}^{N_{p}-N_{c}}B_{0} \end{array}\right]$, and $\Delta U\left(k+1\right)={\left[\begin{array}{cc} \begin{array}{cc} {\Delta u_{m}\left(k+1\right)}^{T} & \cdots \end{array} & {\Delta u_{m}\left(k+N_{c}\right)}^{T} \end{array}\right]}^{T}$, with $2\leq N_{c}\leq N_{p}$. The cost function is defined as

(19) $$J_{n}={\left(R_{m}-Y\left(k+1\right)\right)}^{T}Q\left(R_{m}-Y\left(k+1\right)\right)+\Delta {U\left(k+1\right)}^{T}R\Delta U\left(k+1\right)$$

where $Q={diag\left(\begin{array}{ccccc} k_{id} & k_{iq} & k_{s} & k_{p} & \cdots \end{array}\right)}_{4N_{p}\times 4N_{p}}$ denotes the output weighting matrix, and $k_{id}$, $k_{iq}$, $k_{s}$, and $k_{p}$ are the d-axis current, q-axis current, speed, and position weighting factors, respectively. $R={diag\left(\begin{array}{ccc} k_{u} & k_{u} & \ldots \end{array}\right)}_{\left(2N_{c}-2\right)\times \left(2N_{c}-2\right)}$ denotes the control weighting matrix and $k_{u}$ is the control weighting factor. $R_{m}$ are the reference trajectories of the system outputs, which is expressed as $R_{m}={\left[\begin{array}{ccc} {y^{ref}\left(k+1\right)}^{T} & \cdots & {y^{ref}\left(k+N_{p}\right)}^{T} \end{array}\right]}^{T}$. $y^{ref}\left(k+i\right)=[\begin{array}{cc} i_{d}^{ref}\left(k+i\right) & i_{q}^{ref}\left(k+i\right) \end{array}{\begin{array}{cc} \omega_{e}^{ref}\left(k+i\right) & \theta_{e}^{ref}\left(k+i\right) \end{array}]}^{T}$, where $i_{d}^{ref}$, $i_{q}^{ref}$, $\omega_{e}^{ref}$, and $\theta_{e}^{ref}$ are the d-axis current, q-axis current, angular speed, and angular position reference trajectories, respectively. The $i_{d}=0$ strategy is adopted in this study, so the d-axis current reference trajectory is set to $i_{d}^{ref}\left(k+i\right)=0,\;i\in \left(\begin{array}{ccc} 2 & \cdots & N_{p} \end{array}\right)$. $i_{q}^{ref}$, $\omega_{e}^{ref}$, and $\theta_{e}^{ref}$ are discussed in Section 3.

When the system constraints are not a problem, the unconstrained optimal control sequence $\Delta U_{uc}\left(k+1\right)$ is calculated by minimizing the cost function (19). Substituting (18) into (19) and solving the equality $\partial J_{n}/\partial \Delta U\left(k+1\right)=0$, the closed form of $\Delta U_{uc}\left(k+1\right)$ is obtained as

(20) $$\Delta U_{uc}\left(k+1\right)={\left({\bar{G}}^{T}Q\bar{G}+R\right)}^{-1}{\bar{G}}^{T}Q\left[R_{m}-\bar{F}\bar{x}\left(k\right)\right]$$

## 3. The Proposed ICPS

The unconstrained optimal control sequence $\Delta U_{uc}\left(k+1\right)$ may risk overspeed and overcurrent or exceed the modulation range of the system, so the system constraints must be considered carefully. In this section, the proposed ICPS is presented, where the speed and current constraints are considered within the CTP and the voltage constraint is imposed on $\Delta U_{uc}\left(k+1\right)$ directly.

### 3.1. The Proposed CTP Considering Speed and Current Constraints

The reference trajectories are tracked via the IMS-MPPC algorithm. If these trajectories are speed- and current-constraint-compatible, then it is feasible to ignore these constraints during the optimization. According to the conventional MPC principle, the position trajectory is obtained via smooth processing:

(21) $$\theta_{e}^{ref}\left(k+i\right)=\theta_{e}^{*}-\alpha \left(\theta_{e}^{*}-\theta_{e}^{ref}\left(k+i-1\right)\right)$$

where $\theta_{e}^{*}$ represents the position command. α∈(0,1) is the smooth factor and $i\in \left(\begin{array}{cccc} 2 & 3 & \cdots & N_{p} \end{array}\right)$ is the same as below. Theoretically, the speed trajectory is equal to the first-order difference of $\theta_{e}^{ref}$, that is,

(22) $$\omega_{e}^{ref}\left(k+i\right)=\left(\theta_{e}^{ref}\left(k+i\right)-\theta_{e}^{ref}\left(k+i-1\right)\right)/T_{s}$$

However, the speed trajectory obtained from (22) may not meet the speed constraint, so it should be modified.

(23) $$\omega_{e}^{ref}\left(k+i\right)=\begin{array}{c} \{\begin{array}{ccc} \omega_{emax} & , & \omega_{e}^{ref}\left(k+i\right)>\omega_{emax}\\ -\omega_{emax} & , & \omega_{e}^{ref}\left(k+i\right)<-\omega_{emax}\\ \omega_{e}^{ref}\left(k+i\right) & , & else \end{array} \end{array}$$

where $\omega_{emax}$ is the maximum allowable speed. If the speed limit is triggered at any moment, then the corresponding position trajectory at the same moment should be modified too.

(24) $$\theta_{e}^{ref}\left(k+i\right)=\theta_{e}^{ref}\left(k+i-1\right)+T_{s}\omega_{e}^{ref}\left(k+i\right)$$

To obtain the current trajectory, dynamic (3) is discretized using the backward difference method:

(25) $$i_{q}\left(k\right)=\frac{2J}{3T_{s}p^{2}\psi_{f}}\left(\omega_{e}\left(k\right)-\omega_{e}\left(k-1\right)\right)+\frac{2}{3p\psi_{f}}T_{L}\left(k\right)$$

Defining the constant $\eta =\frac{2J}{3T_{s}p^{2}\psi_{f}}$, the incremental version of (25) can be formulated as follows to weaken the impact of $T_{L}$.

(26) $$\Delta i_{q}\left(k\right)=\eta \left(\Delta \omega_{e}\left(k\right)-\Delta \omega_{e}\left(k-1\right)\right)$$

Then, the current trajectory can be calculated from the speed trajectory according to the following procedure:

(27) $$\Delta \omega_{e}^{ref}\left(k+i\right)=\omega_{e}^{ref}\left(k+i\right)-\omega_{e}^{ref}\left(k+i-1\right)$$

(28) $$\Delta \omega_{e}^{ref}\left(k+1\right)=\omega_{e}\left(k+1\right)-\omega_{e}\left(k\right)$$

(29) $$\Delta i_{q}^{ref}\left(k+i\right)=\eta \left(\Delta \omega_{e}^{ref}\left(k+i\right)-\Delta \omega_{e}^{ref}\left(k+i-1\right)\right)$$

(30) $$i_{q}^{ref}\left(k+i\right)=i_{q}^{ref}\left(k+i-1\right)+\Delta i_{q}^{ref}\left(k+i\right)$$

Similarly, the current trajectory should be modified to meet the current limit.

(31) $$i_{q}^{ref}\left(k+i\right)=\{\begin{array}{ccc} i_{qmax} & , & i_{q}^{ref}\left(k+i\right)>i_{qmax}\\ -i_{qmax} & , & i_{q}^{ref}\left(k+i\right)<-i_{qmax}\\ i_{q}^{ref}\left(k+i\right) & , & else \end{array}$$

where $i_{qmax}$ is the maximum allowable current. If the current limit is triggered at any moment, then the corresponding speed and position trajectories at the same moment should be modified too.

(32) $$\Delta i_{q}^{ref}\left(k+i\right)=i_{q}^{ref}\left(k+i\right)-i_{q}^{ref}\left(k+i-1\right)$$

(33) $$\Delta \omega_{e}^{ref}\left(k+i\right)=\Delta \omega_{e}^{ref}\left(k+i-1\right)+\frac{1}{\eta }\Delta i_{q}^{ref}\left(k+i\right)$$

(34) $$\omega_{e}^{ref}\left(k+i\right)=\omega_{e}^{ref}\left(k+i-1\right)+\Delta \omega_{e}^{ref}\left(k+i\right)$$

(35) $$\theta_{e}^{ref}\left(k+i\right)=\theta_{e}^{ref}\left(k+i-1\right)+T_{s}\omega_{e}^{ref}\left(k+i\right)$$

Formula (35) is identical to (24), rewritten here only for the sake of completeness.

At every control cycle, let $y^{ref}\left(k+1\right)={\left[\begin{array}{cccc} i_{d}\left(k+1\right) & i_{q}\left(k+1\right) & \omega_{e}\left(k+1\right) & \theta_{e}\left(k+i\right) \end{array}\right]}^{T}$, which is determined by the state and control variable in this case. By iteratively calculating from i=2 to $i=N_{p}$ according to (21)–(35), the speed- and current-constraint-compatible reference trajectories $R_{m}$ are produced.

To smoothen the expected trajectory, the generated expected current is averaged to suppress system noise. It is expressed as follows based on the principle of equal impulse:

(36) $$i_{q}^{ref}\left(k+j\right)=\frac{1}{N_{p}-1}\left(\sum_{i=2}^{N_{p}}i_{q}^{ref}\left(k+i\right)\right),\;j=2,\cdots ,N_{p}$$

### 3.2. Voltage Constraint

The voltage constraint is determined by the DC-link voltage and modulation method. Concerning SVPWM technology, the voltage constraint is limited to the voltage vector circle described as

(37) $$u_{q}^{2}\left(k+1\right)+u_{d}^{2}\left(k+1\right)\leq \frac{U_{dc}^{2}}{3}$$

where $U_{dc}$ is the DC-link voltage. According to the principle of receding horizon control, only the first sample, $\Delta u_{m}\left(k+1\right)$, of $\Delta U^{uc}\left(k+1\right)$ is applied to the motor, and the dq-axis control voltages are calculated as

(38) $$\left[\begin{array}{c} u_{d}\left(k+1\right)\\ u_{q}\left(k+1\right) \end{array}\right]=\left[\begin{array}{c} \Delta u_{md}\left(k+1\right)+\rho \\ \Delta u_{mq}\left(k+1\right)+\sigma \end{array}\right]$$

where

$$\rho =u_{d}\left(k\right)-\omega_{e}\left(k+1\right)L_{s}i_{q}\left(k+1\right)+\omega_{e}\left(k\right)L_{s}i_{q}\left(k\right),$$

$$\sigma =u_{q}\left(k\right)+\omega_{e}\left(k+1\right)L_{s}i_{d}\left(k+1\right)-\omega_{e}\left(k\right)L_{s}i_{d}\left(k\right).$$

Let $\sqrt{u_{q}^{2}\left(k+1\right)+u_{d}^{2}\left(k+1\right)}$ be $u_{Amp}$.

(39) $$u_{q}^{*}=\{\begin{array}{c} \frac{u_{q}\left(k+1\right)}{u_{Amp}}\cdot \frac{U_{dc}}{\sqrt{3}},\;u_{Amp}>\frac{U_{dc}}{\sqrt{3}}\\ u_{q}\left(k+1\right),\;else \end{array}$$

(40) $$u_{d}^{*}=\{\begin{array}{c} \frac{u_{d}\left(k+1\right)}{u_{Amp}}\cdot \frac{U_{dc}}{\sqrt{3}},\;u_{Amp}>\frac{U_{dc}}{\sqrt{3}}\\ u_{d}\left(k+1\right),\;else \end{array}$$

Voltage constraints are applied as hard constraints by directly limiting the amplitude after solving the QP problem. In practice, however, the algorithm addresses an unconstrained QP problem with a positive definite weight matrix during the solution process, thereby avoiding deadlock and non-convergence issues.

The control variable ${\left[\begin{array}{cc} u_{d}^{*} & u_{q}^{*} \end{array}\right]}^{T}$ will act on the motor in the next control cycle.

## 4. Extended Luenberger Observer

Due to the presence of noise in actual motor systems, in this study, an extended Luenberger observer is adopted to observe the electrical angle and angular velocity [20]. The form of the observer is as follows:

(41) $$\frac{d\hat{x}}{dt}=A\hat{x}+Bu+Lv$$

where $\;x={\left[\theta_{e},\omega_{e},T_{L}\right]}^{T}$, $v=y-\hat{y}=C(x-\hat{x})$, $L={\left[l_{1},l_{2},l_{3}\right]}^{T}$, and $u=i_{q}$. The notation (^^^) is used to express the estimated variables. The system matrices A, B, and C are defined as follows:

(42) $$A=\left[\begin{array}{ccc} 0 & 1 & 0\\ 0 & 0 & -\frac{p}{J}\\ 0 & 0 & 0 \end{array}\right],\;B=\left[\begin{array}{c} 0\\ \frac{3p^{2}\psi_{f}}{2J}\\ 0 \end{array}\right],\;C=\left[1\;0\;0\right]$$

To simplify the processing, the eigenvalues of matrix (A−LC) are uniformly set to $-\omega_{0}\;(where\;\omega_{0}>0)$. The system is only stable and converges when the eigenvalues have negative real parts. By substituting the characteristic equation det(λI−(A−LC))=0 into the expressions of each matrix in this section, we can derive the equation $\lambda^{3}+l_{1}\lambda^{2}+l_{2}\lambda -\frac{p}{J}l_{3}=0$. Since the eigenvalues of the matrix (A−LC) are all set to $-\omega_{0}$, the characteristic equation of the matrix should be $\lambda^{3}+3\omega_{0}\lambda^{2}+3\omega_{0}^{2}\lambda +\omega_{0}^{3}=0$. Therefore, we can infer that the elements of matrix L are

(43) $$l_{1}=3\omega_{0},\;l_{2}=3\omega_{0}^{2},\;l_{3}=-\frac{{J\omega }_{0}^{3}}{p}$$

## 5. Simulation and Experiment Results

### 5.1. Comparative Simulation Study

A simulation study is conducted to evaluate the performance of the proposed NCCS-MPC in this section, and comparative simulations with MPPC-GC [25] and MPPC-HA are also implemented. Concerning MPPC-HA, it features solving the QP problem (44) and (45) with Hildreth’s algorithm [24].

(44) $$J_{n}={\left(R_{m}-Y\left(k+1\right)\right)}^{T}Q\left(R_{m}-Y\left(k+1\right)\right)+\Delta {U\left(k\right)}^{T}R\Delta U\left(k\right)$$

(45) s.t. MΔU(k)≤γ

When formulating the cost function (44), y(k) is redefined as ${\left[\begin{array}{cc} i_{d}\left(k\right) & \theta_{e}\left(k\right) \end{array}\right]}^{T}$, and therefore the matrices F, G, Q, R, and the reference trajectory $R_{m}$ should be modified accordingly. The constraints (45) can be modeled similarly to [25].

The parameters of the SPMSM used in simulation are listed in Table 1. All the methods are simulated with sampling period $T_{s}=100\;\mu s$ and maximum allowable mechanical angular speed 0.5°/s; that is, the speed constraint is $\omega_{emax}=0.07\;rad/s$. The current constraint is $i_{qmax}=1.2\;A$ for NCCS-MPC and $i_{qmax}=1.2\;A$ and $i_{dmax}=0.5\;A$ for MPPC-GC and MPPC-HA, respectively. Sensor noise is not considered in the simulation. The control parameters of the three methods are determined by trial and error and are listed in Table 2.

**Figure 2:**
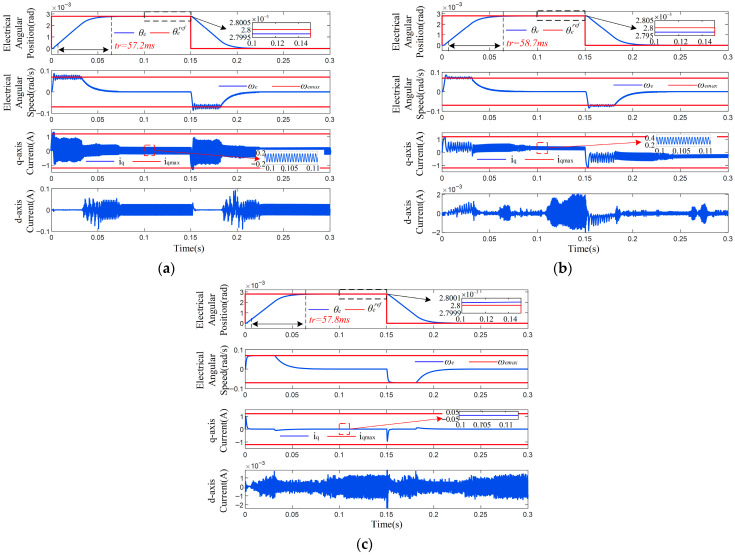
Tracking performance under step input. (**a**) MPPC-HA. (**b**) MPPC-GC. (**c**) NCCS-MPC.

A step signal $\theta_{e}^{*}=2.8\times 10^{-3}\;rad$ is used to test the performance of the three methods, and the response curves are depicted in Figure 2. The rise time, RMS tracking error, q-axis current ripple, and maximum position error during 0.1–0.15 s are measured in Table 3, which imply that the MPPC-GC features the fastest response property but highest static error, and the proposed NCCS-MPC responds faster than MPPC-HA and has the lowest static error. It also proves that the NCCS-MPPC strategy proposed in this paper can effectively reduce waste power and mechanical wear.

More importantly, the following can be seen from the speed and current curves: ① MPPC-GC and MPPC-HA both suffered from oscillation during the transient stage because of the deadlock and non-convergence problem caused by the frequent constraint conflicts, but NCCS-MPC survived thanks to the proposed ICPS, leading to an unconstrained QP problem (19). ② MPPC-GC and MPPC-HA both presented chattering features during the steady state due to the one-step computational delay, but NCCS-MPC survived because this delay was compensated for in the proposed IMS-MPC algorithm. Oscillation and chattering will lead to more waste power and mechanical wear, and the proposed NCCS-MPC strategy can efficiently mitigate these adverse effects.

**Figure 3:**
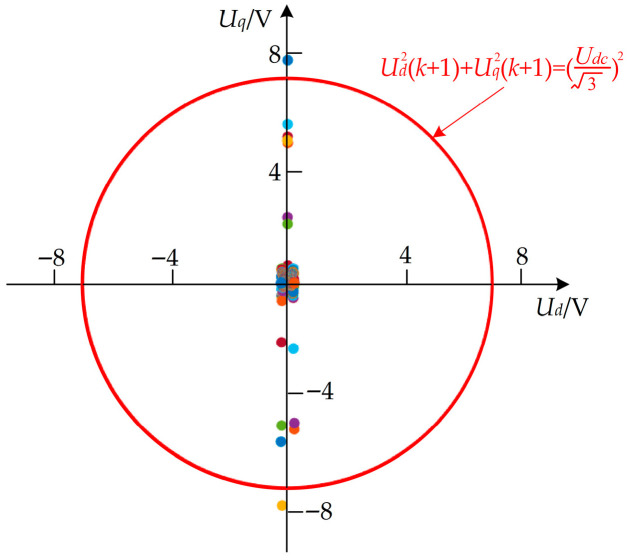
Voltage distribution after solving the QP problem in the simulation.

At the same time, to verify the effectiveness of the algorithm by directly constraining the voltage, Figure 3 shows a plot of the unlimited $u_{d}\left(k+1\right)$ and $u_{q}\left(k+1\right)$ after solving the QP problem. During the simulation, it can be seen that the optimized calculated $\sqrt{u_{q}^{2}\left(k+1\right)+u_{d}^{2}\left(k+1\right)}$ does not exceed the limit in 99.93% of cases, and it will not exceed the limit in the near-steady and steady states; there are very few cases where the limit is exceeded only in the transient response process. In this case, the d- and q-axis voltage after the limit is not the optimal solution; only the amplitude has been reduced, which does not affect the overall transient performance.

### 5.2. Experimental Result

**Figure 4:**
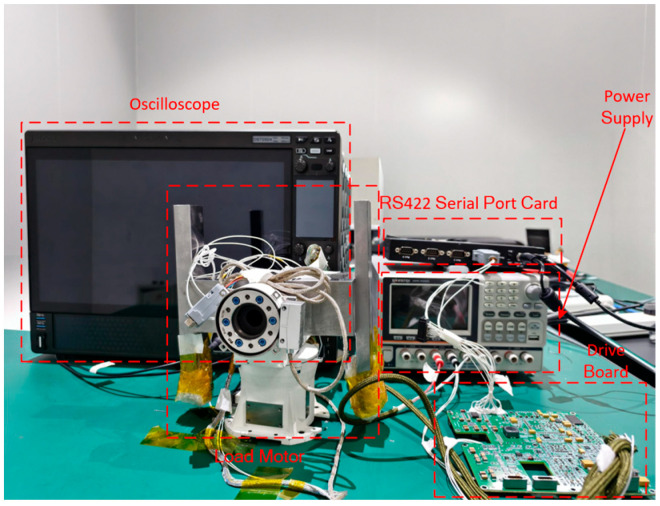
Experimental setup.

To verify the practical effectiveness of the proposed NCCS-MPC strategy and its consistency with the simulation conclusions, experiments are conducted on a set of high-performance SPMSM experimental platforms. A 26-bit absolute encoder is selected, and the PWM wave carrier frequency is 10 KHz. The motor parameters are completely consistent with those in Table 1, and the experimental period, trajectory constraints, etc., are consistent with the simulation to ensure the comparability of the experimental and simulation conditions. The experimental platform is shown in Figure 4.

**Figure 5:**
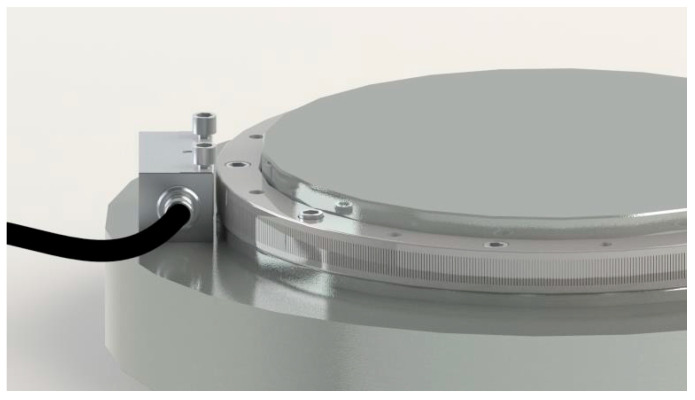
Metal grating angle encoder.

According to the size and precision requirements of the rotating shaft, a metal grating angle encoder is adopted. With image acquisition and processing technology as the core, a signal super-resolution decoding method is used for high-precision and miniaturized angle sensing measurement. It has excellent characteristics such as a large hollow shaft, light weight, and small rotational inertia, and it is suitable for controlling hollow shaft structure turntables. Its split structure design allows the encoder disk to be directly fixed on the rotating component, reducing measurement errors. The main technical parameters are shown in Table 4, and Figure 5 shows an image of the device.

**Figure 6:**
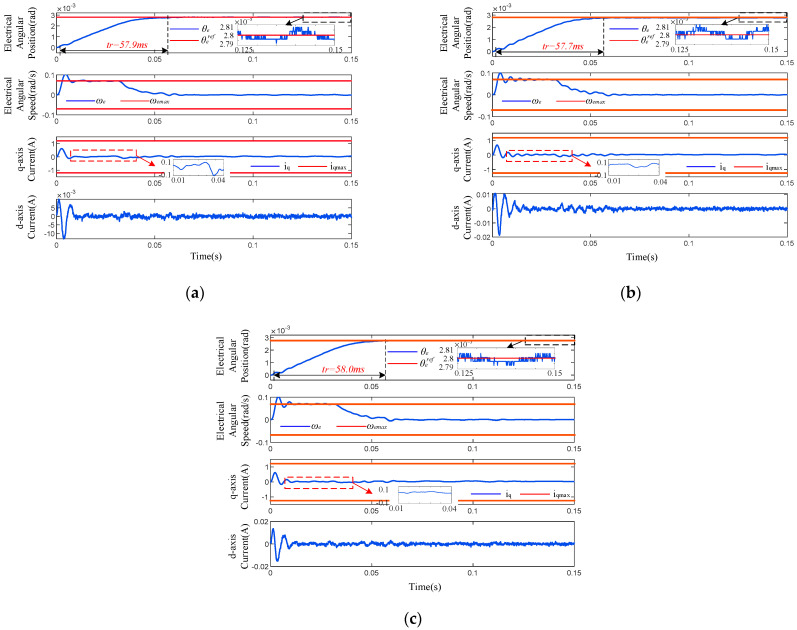
Tracking performance under step input. The deviation of model parameters is (**a**) 0, (**b**) 20%, and (**c**) −20%.

A step position instruction with an amplitude of $\theta_{e}^{*}=2.8\times 10^{-3}$ is applied to the system. Figure 6a shows the experimental results from adopting the proposed NCCS-MPC strategy. It can be observed that the motor tracks the instruction rapidly and accurately, and the position transient response is smooth and without overshoot. The rise time is 57.9 ms and the maximum steady-state error is less than 10 μrad. At the same time, to prove the feasibility of using a control frequency of 10 kHz, the actual time taken for the control algorithm to execute one cycle was measured to be 65.6 μs, which is less than 100 μs and meets the requirement. The speed and current responses are smooth during the transient process, and they are confined within the preset constraint boundaries (indicated by the red line in the figure) after a brief overjump, which fully demonstrates the effectiveness of the ICPS in handling constraints, and the IMS-MPPC algorithm successfully compensates for the computational delay.

Parameters such as the resistance and inductance of the SPMSM system are clearly provided by the motor, while the moment of inertia depends on the load and the measurement method is limited. When the actual moment of inertia of the SPMSM system deviates from the nominal value, the system model parameters will not match. Experiments were conducted to verify the robustness of the algorithm: the moment of inertia parameters were modified in the software program since the actual load was immutable. The experimental results are shown in the figure. Figure 6b,c show the response curves when the nominal moment of inertia deviates by 20% and −20%, respectively, indicating that the transient response time and steady-state error barely change.

**Figure 7:**
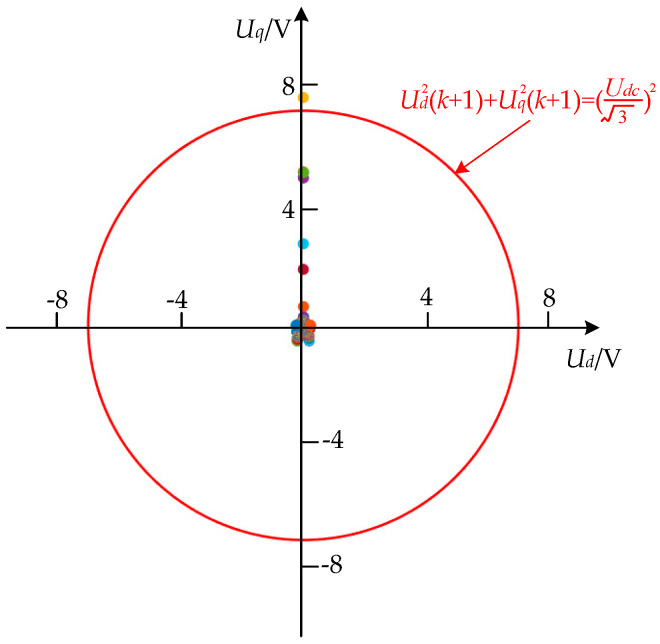
Voltage distribution after solving the QP problem in the experiment.

After solving the QP problem in the simulation, the d- and q-axis voltage distribution prior to application of the voltage hard constraint was analyzed. To verify the consistency in an actual motor control system, the above voltage values were measured, and the distribution is shown in Figure 7. The same conclusions as in the simulation can be drawn.

According to the experiment, the core performance of the algorithm was reproduced in the experimental environment, the rise time was highly consistent, and the robustness of the model parameters was good. However, the steady-state error increased, which can be attributed to the non-ideal factors of the actual system, such as mechanical friction, encoder noise, inverter dead zone, and parameter uncertainty. However, it still maintains a high precision at the milliradian level and demonstrates good and stable transient performance in the experiment.

The experimental results are highly consistent with the simulation predictions, which strongly proves that the proposed NCCS-MPC strategy is not limited to the simulation environment. It significantly improves the dynamic performance, steady-state performance, and reliability of the system, meeting the requirements of high-precision industrial applications.

## 6. Conclusions

In this paper, we propose a strategy for novel continuous control set model predictive control (NCCS-MPC) for the high-performance position control problem of an SPMSM. This strategy is organically composed of two parts: the improved multi-step model predictive position control (IMS-MPPC) algorithm and the innovative constraint handling strategy (ICPS). It can achieve high-precision fast responses and overshoot position control in both simulation environments and real systems. At the same time, it can properly handle multi-constraint problems and eliminate the adverse effects caused by computational delays. The combination of the two strategies not only ensures good performance in steady-state tracking but also addresses the issues caused by constraint conflicts. Although the solution obtained by directly using series voltage limiting after solving the unconstrained QP problem may not be optimal, under actual working conditions, the limiting conditions are met in both the steady state and when the transient response is over 99.9%, and it will not affect the control of the system. In conclusion, the NCCS-MPC strategy proposed in this paper provides an effective solution for controlling the SPMSM with high precision and reliability, and has broad application prospects in high-end fields such as industrial robots, precision optical instruments, and semiconductor manufacturing equipment.

## Figures and Tables

**Table 1:** Parameters of SPMSM.

Symbol	Quantity	Value
$L_{s}$	Stator dq-axis inductance	1.8 mH
$R_{s}$	Stator phase resistance	2.5 Ω
$\psi_{f}$	Permanent magnetic flux linkage	0.016 Wb
J	Moment of inertia	0.0268 kg∙m^2^
p	Pole pairs	8
$U_{dc}$	DC-link voltage	12 V
$f_{s}$	Viscous friction coefficient	4.39 × 10^−3^ N∙m∙s

**Table 2:** Control parameters of SPMSM.

Items	MPPC-HA	MPPC-GC	NCCS-MPC
Position weighting factor, $k_{p}$	1	1	1
Speed weighting factor, $k_{s}$	*	*	1
q-axis current weighting factor, $k_{iq}$	*	*	1
d-axis current weighting factor, $k_{id}$	1	1	1
Control weighting factor, $k_{u}$	0	0	0.01
Predictive horizon, $N_{p}$	10	10	10
Control horizon, $N_{c}$	2	2	2
Smooth factor, α	0.99	0.99	0.99

The * in the table indicates that there is no parameter.

**Table 3:** Comparison of control effects.

Items	MPPC-HA	MPPC-GC	NCCS-MPC
Position rise time (ms)	57.2	58.7	57.8
Maximum static position error (μrad)	0.33	3.54	0.05
RMS tracking error (μrad)	0.002	0.008	0.0002
Q-axis current ripple (A)	0.18	0.3132	$2.12\times 10^{-4}$

**Table 4:** Raster model parameters.

Outer Diameter (mm)	Resolution	Communication Interface	Voltage (V)	Mass (kg)
52	26 bit	BiSS_C	5	0.1

## Data Availability

The raw data supporting the conclusions of this article will be made available by the authors on request.
